# Development and validation of a prognostic nomogram for predicting in-hospital mortality of patients with acute paraquat poisoning

**DOI:** 10.1038/s41598-023-50722-z

**Published:** 2024-01-18

**Authors:** Guo Tang, Zhen Jiang, Lingjie Xu, Ying Yang, Sha Yang, Rong Yao

**Affiliations:** Emergency Medicine Laboratory and the Department of Emergency, West China Hospital, Sichuan University, No. 37 Guoxue Alley, Chengdu, 610041 Sichuan China

**Keywords:** Health occupations, Medical research, Risk factors

## Abstract

This study aimed to develop and validate a predictive model to determine the risk of in-hospital mortality in patients with acute paraquat poisoning. This retrospective observational cohort study included 724 patients with acute paraquat poisoning whose clinical data were collected within 24 h of admission. The primary outcome was in-hospital mortality. Patients were randomly divided into training and validation cohorts (7/3 ratio). In the training cohort, the least absolute shrinkage and selection operator regression models were used for data dimension reduction and feature selection. Multivariate logistic regression was used to generate a predictive nomogram for in-hospital mortality. The prediction model was assessed for both the training and validation cohorts. In the training cohort, decreased level of consciousness (Glasgow Coma Scale score < 15), neutrophil-to-lymphocyte ratio, alanine aminotransferase, creatinine, carbon dioxide combining power, and paraquat plasma concentrations at admission were identified as independent predictors of in-hospital mortality in patients with acute paraquat poisoning. The calibration curves, decision curve analysis, and clinical impact curves indicated that the model had a good predictive performance. It can be used on admission to the emergency department to predict mortality and facilitate early risk stratification and actionable measures in clinical practice after further external validation.

## Introduction

Paraquat (1,1′-dimethyl-4,4′-bipyridinium dichloride [PQ]) is a non-selective contact herbicide widely used in many countries since the 1960s^1^. Acute PQ poisoning (APP) has been associated with suicides and accidents, with PQ causing sequential organ damage/failure through oxidative stress and systemic inflammatory responses. The most prominent manifestations include acute lung and kidney injuries^2^. The mortality rate of patients with APP remains tremendously high (50–90%)^3–6^. Consequently, PQ has been banned in most countries, including China. However, PQ is still legally used in some areas^7,8^.

Early assessment of the severity and prognosis of patients with APP is crucial to guide treatment (hemodilution, immunosuppressive therapy)^9,10^. Currently, no standardized method exists for predicting the prognosis of APP. Previous studies have demonstrated the utility of the Severity Index of PQ Poisoning (SIPP) as a specific scoring system for prognosticating APP^11^. However, calculating the SIPP score necessitates the measurement of serum PQ concentration, which demands costly equipment. Consequently, its usage is limited in most countries, particularly in developing nations^12,13^. Furthermore, research indicates that the SIPP score might overestimate the survival rate of patients with APP^14^, especially those admitted to the hospital more than 24 h after poisoning^15^. Prior research has identified complete blood cell count^16^, liver and kidney function indicators^17^, serum anion gap^18,19^, and chest computed tomography^20^ as independent prognostic indicators for APP. Nevertheless, these indicators are relatively individualistic and demonstrate limited predictive power. The currently available scoring systems for predicting patient prognosis, including the Acute Physiology and Chronic Health Evaluation II (APACHE II) score^21^, Sequential Organ Failure Assessment (SOFA) score^22^, and Poisoning Severity Score (PSS)^23^, are designed for critically ill patients rather than individuals with low exposure or mild symptoms.

Furthermore, due to the intricate calculation process, these scoring systems are incapable of promptly predicting the mortality rate or conducting risk assessments for PQ poisoning patients^24^. Accordingly, it is imperative to develop an efficient, straightforward, and universally applicable predictive model based on commonly employed laboratory indicators. A nomogram is a simple multivariate prediction model that incorporates multiple variables affecting prognosis to calculate an individual’s survival probability^25^. Therefore, this study aims to develop a nomogram model based on clinical data collected within 24 h of admission that can identify, at an early stage, patients who are at high risk of in-hospital mortality due to APP.

## Methods

### Setting

We performed a retrospective, observational cohort study in an emergency department (ED) with more than 3000 beds in an extensive tertiary care teaching hospital in Chengdu City, Sichuan Province, China, per the amended Declaration of Helsinki. The West China Hospital approved the study of the Sichuan University Biomedical Research Ethics Committee (No. 2022-1591). Due to the retrospective nature of the study, the need of informed consent was waived by the Sichuan University Biomedical Research Ethics Committee. All analyses were performed following the Transparent Reporting of a multivariate prediction model for Individual Prognosis or Diagnosis statement^26^.

### Patients

We conducted a study involving patients diagnosed with APP who were admitted to the ED of West China Hospital, Sichuan University, between September 1, 2010, and January 31, 2022. Inclusion criteria comprised of patients aged ≥ 14 years, those with poisoning via gastrointestinal intake, and individuals with PQ plasma concentrations ≥ 0.01 mg/L by high-performance liquid chromatography. Exclusion criteria encompassed patients whose PQ concentrations were not detected in the plasma, those with incomplete clinical data, those with other concomitant poisoning (such as alcohol poisoning), and pregnant women.

For patients who developed symptoms within 6 h of onset, gastric lavage was recommended. Those who developed symtoms within 12 h of onset were administered activated charcoal via oral or nasogastric route. Patients with positive concentrations of PQ in blood tests were advised to undergo blood purification treatment, including blood perfusion, hemodialysis, or continuous venovenous hemofiltration. After excluding contraindications such as gastrointestinal bleeding, all patients should be given methylprednisolone sodium succinate at a dosage of 80 mg/day for anti-inflammatory treatment through intravenous infusion^27^.

### Predictors

We collected anonymous clinical data within 24 h of admission to the Emergency Department (ED) from electronic medical records. The collected data comprised the following variables: time from poisoning to treatment at the study site, sex, age, level of consciousness (LOC), heart rate (HR), respiration rate (RR), systolic blood pressure (SBP), diastolic blood pressure (DBP), white blood cell (WBC) count, neutrophil-to-lymphocyte ratio (NLR), monocyte-to-lymphocyte ratio (MLR), platelet count (PLT), total bilirubin, alanine aminotransferase (ALT), aspartate aminotransferase (AST), alkaline phosphatase (ALP), blood urea nitrogen (BUN), creatinine, cystatin C, carbon dioxide combining power (CO2CP), blood potassium, plasma PQ concentrations, and SIPP. Carbon dioxide combining power (CO2CP) measures a substance or solution's ability to react with and bind carbon dioxide (CO_2_) molecules. It quantifies the substance's capacity to combine with CO_2_ chemically. The normal range of CO2CP typically falls between 22 and 30 milliequivalents per liter (mEq/L)^28^. Decreased LOC is defined as a Glasgow Coma Scale (GCS) score below 15^29^. The severity of PQ poisoning was estimated quantitatively by using the SIPP, which was calculated by multiplying the elapsed time (hours) from ingestion to arrival by the serum PQ level(μg/ml)^11^. All patient data were anonymized and de-identified. Variables were treated as continuous variables, except for sex and disturbance of consciousness, which were binary variables. Finally, the continuous variables used to construct the nomogram were transformed into categorical variables based on the actual data. This was achieved by categorizing them based on the normal upper and lower bounds, median, tertiles, and quartiles.

### Clinical outcome

The patients were divided into two groups: survivor and non-survivor group, according to the occurrence of in-hospital death. The in-hospital mortality discussed in this study refers to all-cause mortality of APP patients, including deaths in the ED.

### Statistical analysis

Data were analyzed using R version 4.2.1 (R Foundation for Statistical Computing, Vienna, Austria). A two-sided *P* value < 0.05 indicated statistical significance. Data are presented as medians (interquartile ranges) for continuous variables and as numbers (percentages) for categorical variables, as appropriate. The non-parametric Mann–Whitney U test, chi-square analysis, and Fisher’s exact test were used to test for differences between groups, as appropriate. In this study, grouping was achieved through simple random non-relaxation sampling using the simple_ra() function in the R software. All the baseline characteristics were comparable between the training and validation cohorts.

In the training cohort, the least absolute shrinkage and selection operator (LASSO) regression was used to decrease the potential collinearity of variables assessed from the same patient and the overfitting of variables. The latent variables in the LASSO regression analysis were included in the multivariate analysis of stepwise forward selection to determine independent risk factors affecting in-hospital mortality. The results are reported as odds ratios (ORs) and 95% confidence intervals (95% CI). A simple nomogram based on the independent risk factors was developed to predict the probability of mortality. The prediction model was assessed using the concordance index (C-index), area under the receiver operating characteristic (ROC) curve (AUC), calibration curves, decision curve analysis (DCA), and clinical impact curves for both the training and validation cohorts^30–32^.

## Results

### Patients

**Figure 1:**
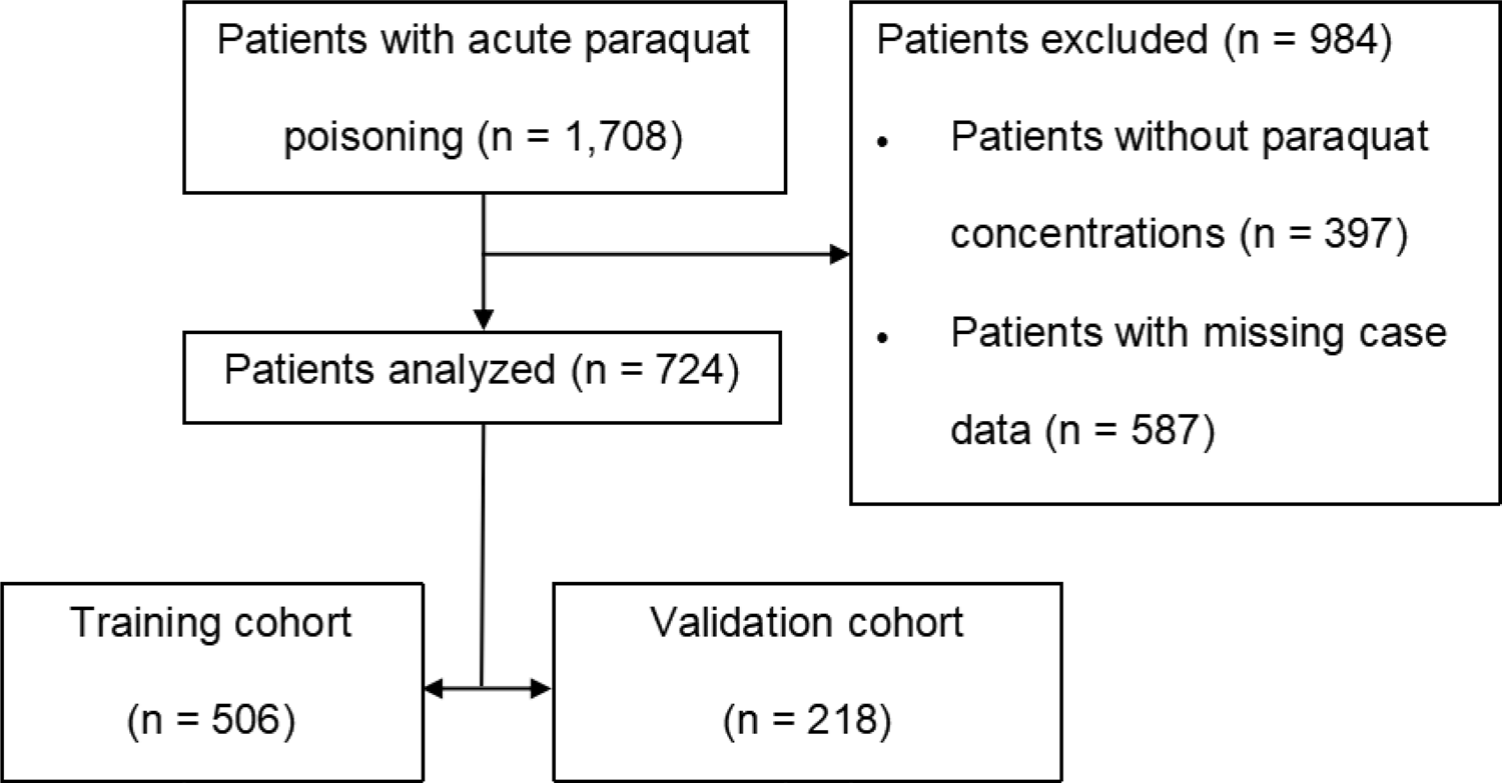
Flowchart of the study.

A total of 1708 patients with APP presented to our ED, of whom 984 were excluded. Finally, 724 patients who met the inclusion criteria in this study were randomly divided into two groups: training and validation cohorts group using a 7:3 ratio (Fig. 1); among them, 360/724 patients (49.7%) died. In terms of demographics, there were 325/724 (44.9%) males, with a median age of 33 (23–42) years. Among 506/724 patients in the training group, 44.1% were male, with a median age of 29 (22–42) years, and 253/506 (50%) patients died. Of the 218/724 patients in the validation group, 46.8% were male, with a median age of 31 (23–42) years, and 107/218 (49.1%) patients died. No statistical significant differences were observed between the two groups (*P* > 0.05) (Table 1).

**Table 1:** Comparison of baseline characteristics between the training and validation cohorts.

Risk factors	Training cohort (n = 506)	Validation cohort (n = 218)	*P*
Time from poisoning to treatment at the study site (h)	6.00 [3.00, 18.0]	7.00 [3.00, 18.2]	0.760
Sex (male)	223 (44.1)	102 (46.8)	0.500
Age (years)	29 [22,42]	31 [23,42]	0.398
Decreased LOC(GCS < 15)	47 (9.29)	16 (7.34)	0.394
Heart rate (times/min)	84 [74.2, 93]	84 [73, 94]	0.664
Respiration rate (times/min)	20 [20,22]	20 [20,22]	0.941
Systolic blood pressure (mmHg)	120 [111, 132]	118 [109, 128]	0.143
Diastolic blood pressure (mmHg)	75.0 [68.0, 81.8]	74.5 [66.2, 82.0]	0.675
White blood cells (× 10^9^/L)	13.6 [9.67, 20.4]	13.1 [8.67, 19.2]	0.373
Neutrophil-to-lymphocyte ratio	11.0 [6.30, 18.2]	9.66 [5.26, 17.1]	0.142
Monocyte-to-lymphocyte ratio	0.42 [0.25, 0.72]	0.39 [0.23, 0.63]	0.129
Platelets (× 10^12^/L)	176 [135, 223]	168 [134, 219]	0.540
Total bilirubin (μmol/L)	13.3 [10.0, 18.9]	13.4 [9.50, 20.8]	0.838
Alanine aminotransferase (IU/L)	21.0 [15.0, 34.0]	22.0 [16.0, 38.0]	0.136
Aspartate aminotransferase (IU/L)	27.0 [21.0, 39.0]	28.0 [22.0, 43.8]	0.246
Alkaline phosphatase (IU/L)	77.0 [61.2, 97.0]	77.0 [62.0, 99.8]	0.889
Blood urea nitrogen (mmol/L)	5.96 [4.51, 7.81]	5.97 [4.51, 7.77]	0.832
Creatinine (μmol/L)	99.0 [71.0, 176]	93.0 [70.0, 190]	0.747
Cystatin C (mg/L)	0.76 [0.65, 0.96]	0.80 [0.66, 1.01]	0.146
CO_2_CP (mmol/L)	18.5 [14.4, 21.5]	18.6 [14.5, 21.1]	0.977
Blood potassium (mmol/L)	3.20 [2.77, 3.56]	3.28 [2.79, 3.68]	0.204
Plasma PQ concentrations (mg/L)	1.63 [0.34, 11.6]	1.64 [0.35, 12.9]	0.870
Mortality	253 (50.0)	107 (49.1)	0.821

Values are presented as medians [interquartile ranges] or n (%).

*LOC* level of consciousness, *GCS* glasgow coma scale, *CO*_*2*_*CP* carbon dioxide combining power, *PQ* paraquat.

### Model development

**Figure 2:**
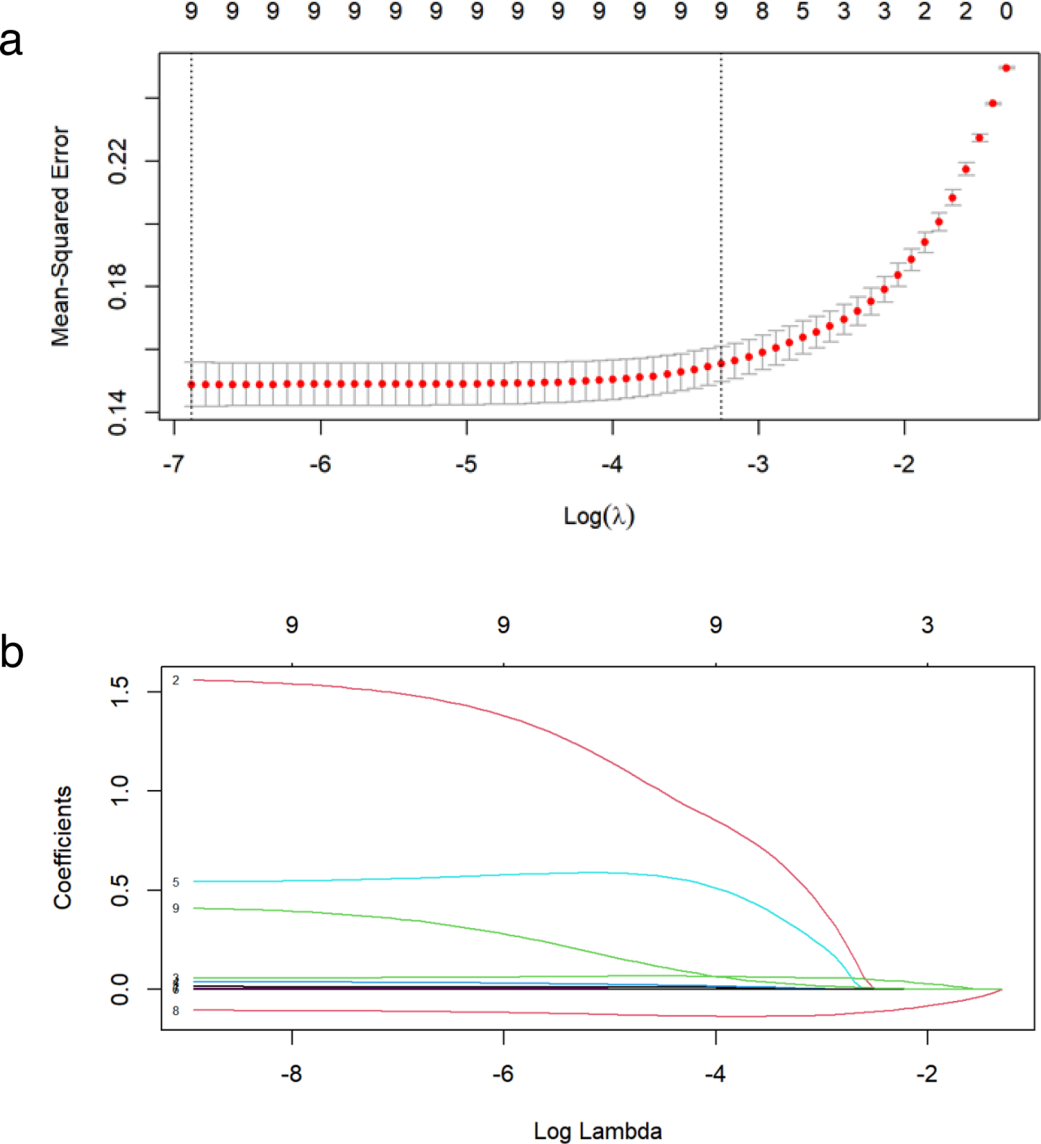
Demographic and clinical feature selection using the LASSO binary logistic regression model. (**a**) Optimal parameter (λ) selection in the LASSO model used 10-fold cross-validation via the minimum criteria. A partial likelihood deviance (binomial deviance) curve was plotted against log(λ). Dotted vertical lines are drawn at the optimal values using the minimum criteria and 1SE of the minimum criteria (1-SE criteria). (**b**) LASSO coefficient profiles of nine features. A coefficient profile plot was constructed against log(λ) sequence. A vertical line was drawn at the value selected using 10-fold cross-validation, where the optimal lambda resulted in eight features with non-zero coefficients. *LASSO* least absolute shrinkage and selection operator, *SE* standard error.

In the demographic and clinical characteristics analysis of 506 patients, 22 features were reduced to nine potential predictive features. These features had non-zero coefficients in the LASSO regression model, which included age, LOC, WBC count, NLR, MLR, ALT, creatinine, CO_2_CP, and plasma PQ concentrations (Fig. 2a,b).

### Construction of nomogram

Multivariate analysis revealed several independent risk factors for in-hospital mortality upon admission, including decreased LOC, NLR, ALT, creatinine, CO_2_CP, and plasma PQ concentrations. The detailed ORs and 95% CI of the multivariate analysis are summarized in Table 2.

**Table 2:** Risk factors for in-hospital mortality in patients with acute paraquat poisoning.

Variable	Crude OR (95% CI)	Adjusted OR (95% CI)	P (Wald’s test)	P (LR test)
Decreased LOC(GCS < 15)	9.87 (3.84–25.41)	4.69 (1.3–16.85)	0.018	0.016
White blood cell (× 10^9^/L)	1.2 (1.16–1.25)	1.06 (0.99–1.13)	0.095	0.093
Neutrophil-to-lymphocyte ratio	1.07 (1.05–1.1)	1.05 (1.01–1.09)	0.014	0.011
Alanine amino transferase (IU/L)	1.0029 (1.0006–1.0052)	1.0024 (0.9998–1.0049)	0.07	0.037
Creatinine (μmol/L)	1.0038 (1.0023–1.0053)	1.002 (1.0004–1.0037)	0.017	0.016
Carbon dioxide combining power	0.74 (0.7–0.78)	0.91 (0.84–0.98)	0.013	0.012
Plasma paraquat concentrations (mg/L)	1.68 (1.49–1.9)	1.55 (1.36–1.76)	< 0.001	< 0.001

*OR* odds ratio, *CI* confidence interval, *LOC* level of consciousness, *GCS* glasgow coma scale, *LR* likelihood ratio.

**Figure 3:**
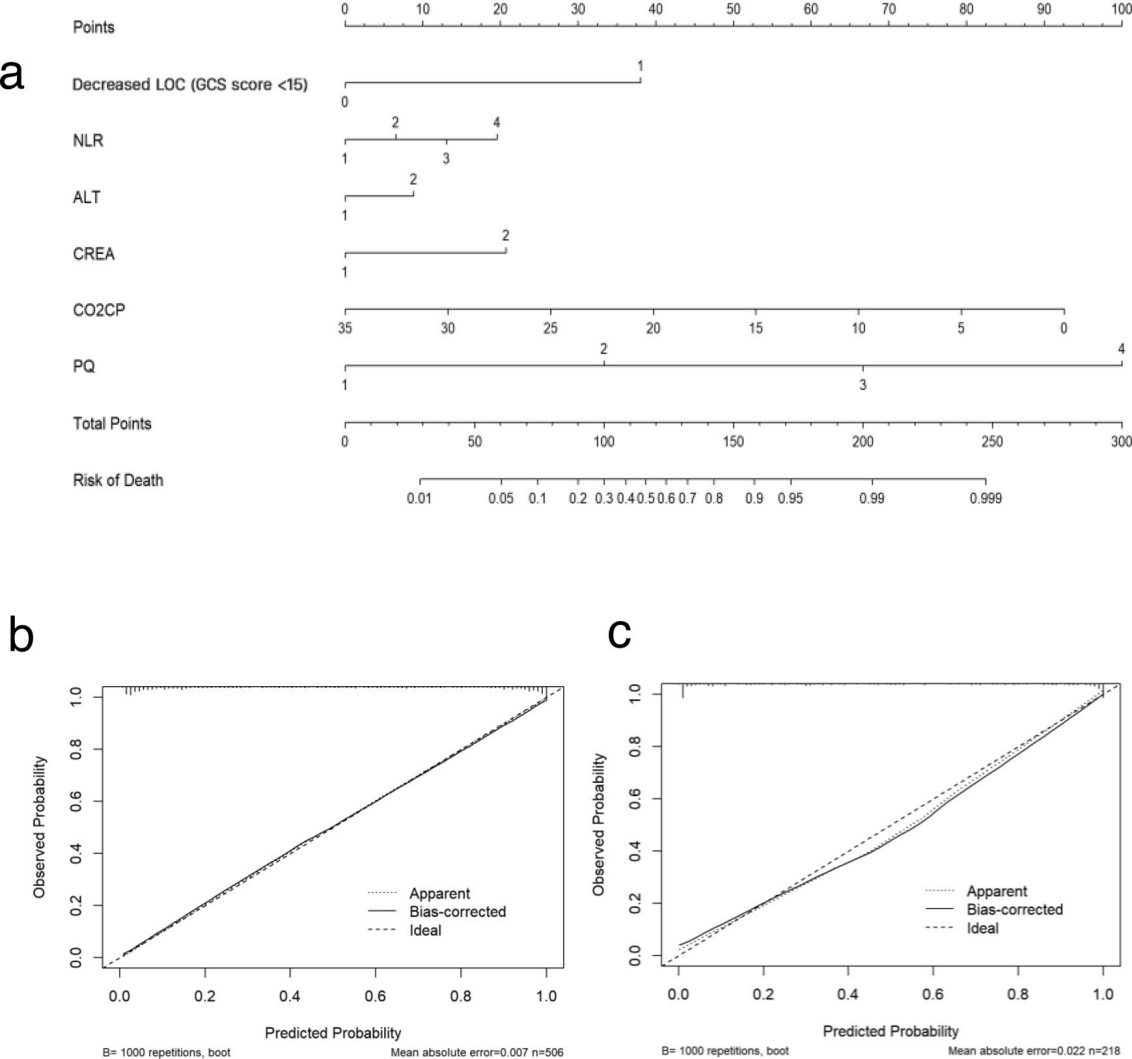
**(a)** Nomogram for in-hospital mortality in patients with acute paraquat poisoning. Decreased LOC (GCS score < 15) (0, no; 1, yes), NLR (1, < 6.2; 2, 6.2–10.94; 3, 10.94–18.02; 4, > 18.02), ALT (1, 0–39; 2, ≥ 40 IU/L), CREA (1, ≤ 110; 2, > 110 µmol/L), PQ (1, ≤ 0.33; 2, 0.33–1.66; 3, 1.66–11.15; 4, ≥ 11.15 g/mL). (**b**) Calibration curve of the nomogram in the training set. (**c**) Calibration curve in the validation set. *LOC* level of consciousness, *GCS* glasgow coma scale, *NLR* neutrophil-to-lymphocyte ratio, *ALT* alanine aminotransferase, *CREA* creatinine, *CO*_*2*_*CP* carbon dioxide combining power, *PQ* paraquat.

Therefore, these six factors were used to construct the prediction model, as shown in Table 2. A simple nomogram was developed to predict the probability of death based on the independent risk factors (Fig. 3a). The NLR values were categorized into quartiles as follows: category 1 for NLR < 6.2, category 2 for NLR 6.2–10.94, category 3 for NLR 10.94–18.02, and category 4 for NLR > 18.02. ALT values were categorized based on the upper limit of normal as category 1 for ALT 0–39 IU/L and category 2 for ALT ≥ 40 IU/L. Additionally, categorical variables were created based on the median value of creatinine, resulting in category 1 for creatinine ≤ 110 μmol/L and category 2 for creatinine > 110 μmol/L. Finally, PQ values were categorized into quartiles: category 1 for PQ ≤ 0.33 g/mL, category 2 for PQ 0.33–1.66 g/mL, category 3 for PQ 1.66–11.15 g/mL, and category 4 for PQ > 11.15 g/mL. As shown in the nomogram, each factor was assigned a point, and the total nomogram points were calculated by summing the individual points of all predictors. The relationship between the total points and the probability of death is presented at the bottom of the nomogram. As an example, CO2CP was considered as a continuous variable. Where a decrease of 5 units corresponded to an approximate 35 points increase in the risk score. The nomogram depicts the predicted probability of in-hospital death resulting from APP, measured on a scale of 0 to 300. Draw a vertical line upward and assign labels to signify the respective points for each covariate. Repeated this procedure for each covariate to ascertain the cumulative.

### Assessment of nomogram

The calibration curve in Fig. 3b,c suggests high consistency, demonstrating that the model’s predicted probabilities are close to the observed actual probabilities. The bias-corrected C-indices for the training and validation cohorts were 0.933 and 0.947, respectively.

**Figure 4:**
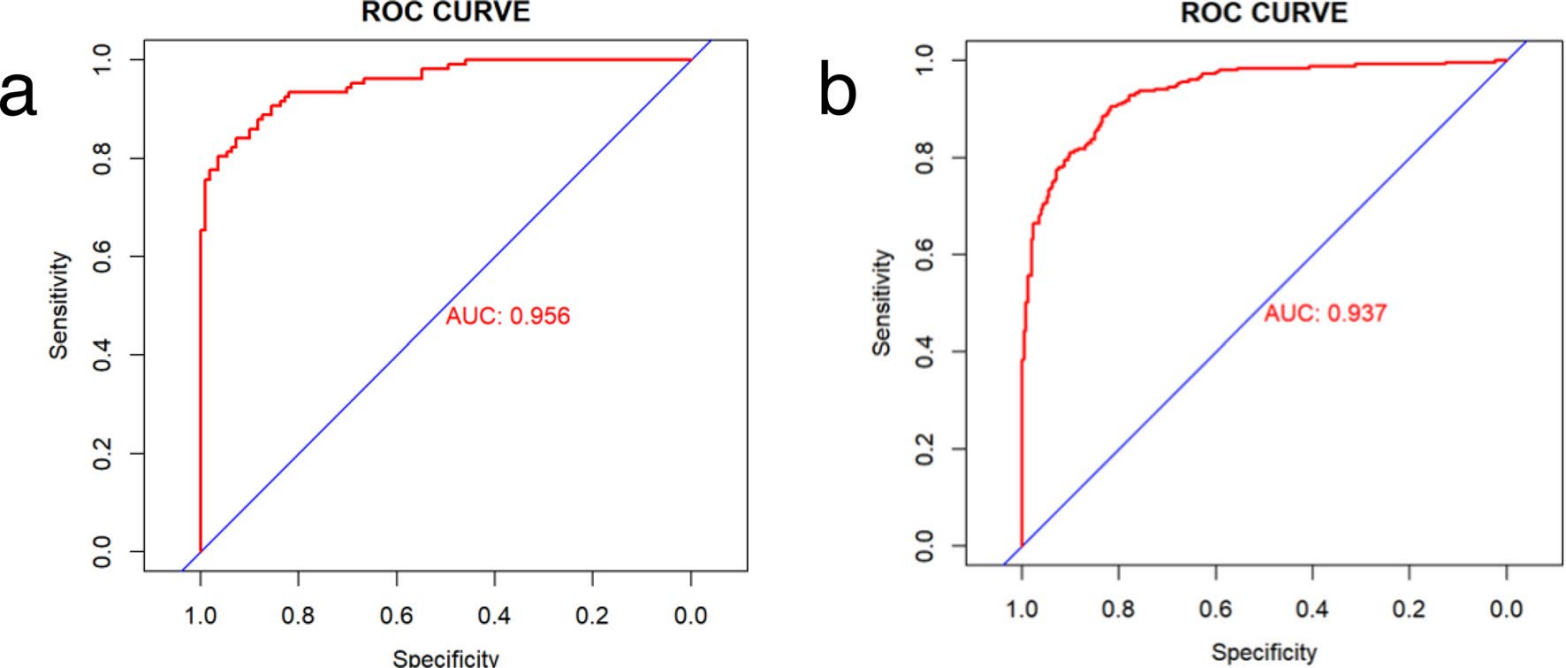
ROC curve of the nomogram for the training (**a**) and validation (**b**) cohorts. *ROC* receiver operating characteristic, *AUC* area under the ROC curve.

**Figure 5:**
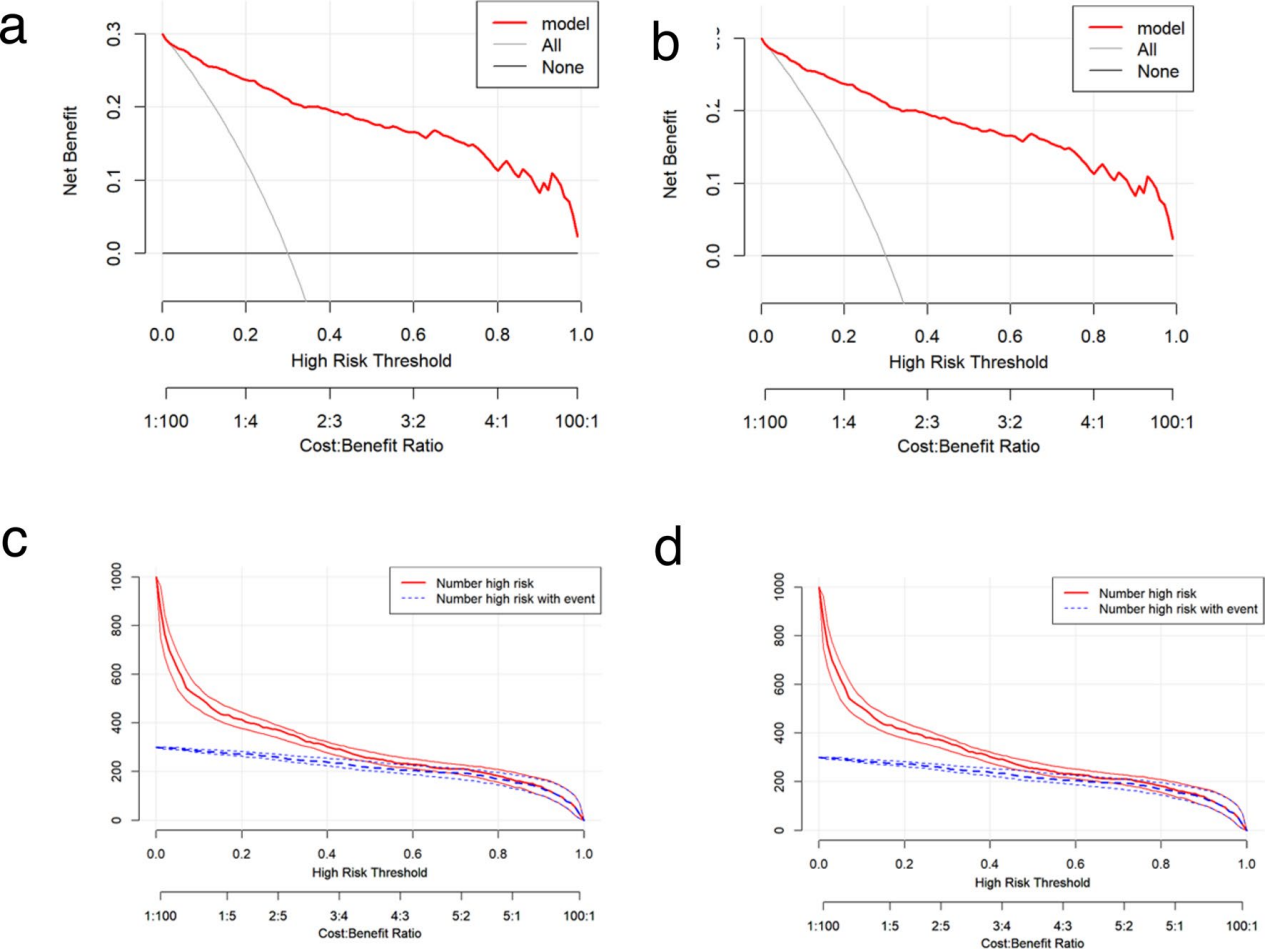
Nomogram decision curve analysis for the training (**a**) and validation (**b**) cohorts, and clinical impact curves for the training (**c**) and validation (**d**) cohorts.

In the training and validation cohorts, the C-indices were 0.953 (95% CI 0.936–0.970) and 0.947 (95% CI 0.920–0.974), respectively, indicating excellent accuracy. The ROC curves and AUC for the training and validation cohorts are shown in Fig. 4a,b. The DCA demonstrated that the nomogram had a superior overall net benefit across a wide range of practical threshold probabilities (Fig. 5a,b). In addition, we plotted clinical impact curves to predict improved probability stratification for a population size of 1000. The predicted probability coincided with the actual probability in the training and validation cohorts (Fig. 5c,d).

### Construction and comparison of other predictive models

**Figure 6:**
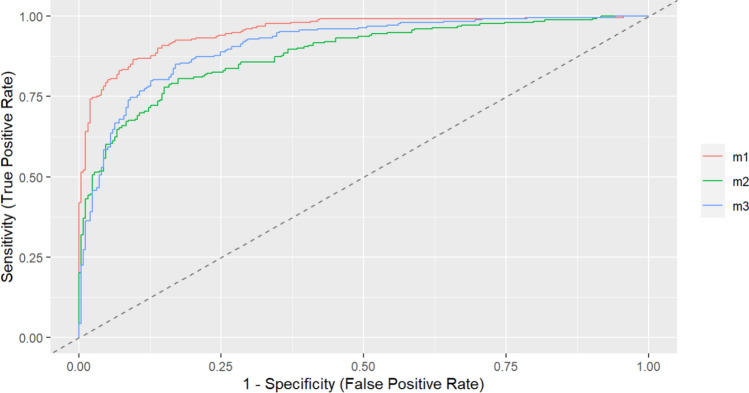
Comparison of other predictive models. m1, nomogram of predictive models, AUC: 0.953 (95% CI 0.936–0.970); m2, predictive model excluding plasma PQ concentrations, AUC: 0.882 (95% CI 0.853–0.911); m3, SIPP, AUC: 0.909 (95% CI 0.883–0.934); *AUC* area under the receiver operating characteristic curve, *SIPP* severity index of paraquat.

Since plasma PQ concentrations accounted for much of the model and some primary hospitals could not detect them, we used LASSO and multivariate logistic regression to construct another model without PQ plasma concentrations. Finally, we determined the following independent risk factors: LOC, age, WBC count, MLR, ALT Plasma PQ concentrations, and CO_2_CP. Concurrently, we included the SIPP to compare the effectiveness of the three prediction models and construct the ROC curve (Fig. 6).

## Discussion

Timely identification of the severity of patients with PQ poisoning is of utmost importance in clinical practice. It assists in formulating targeted treatment plans, allocating medical resources efficiently, and potentially enhancing patient prognosis. Nevertheless, currently, no universally accepted method exists to comprehensively evaluate the correlation between clinical indicators and the survival outcomes of patients with APP. This study bridges this gap by developing a prediction model that evaluates the risk of in-hospital mortality using clinical data collected during admission. The created nomogram exhibited exceptional performance in both the training and validation cohorts, with an AUC exceeding 0.9. The included indicators can be readily acquired upon admission, and the nomogram score can be calculated with ease. These characteristics render the model appropriate for swift and uncomplicated clinical implementation, facilitating early-stage evaluation and prognosis prediction. Its convenience further encourages widespread adoption. Additionally, based on our comprehension, the sample size employed in this study is one of the largest worldwide, and the constructed model has achieved near-perfect prediction performance. Moreover, after comparing the nomogram to SIPP, we have ascertained the superiority of our prediction model.

Prior nomogram models developed to predict the prognosis of APP patients primarily originated from the research conducted by Lu Shan. They retrospectively included a total of 80 cases of patients with APP, utilizing serological markers and imaging examinations to construct their nomogram. The AUC in their training set and validation set were 0.953 (95% CI 0.936–0.970) and 0.947 (95% CI 0.920–0.974), respectively. In comparison to their study, our current study encompasses an expanded sample size, a wider array of accessible indicators and demonstrates exceptional predictive capacity.

Our study revealed a high mortality rate in patients with decreased LOC due to APP, possibly related to toxic encephalopathy or hyperemia. Previous research has reported that PQ can stimulate glutamate efflux, leading to excitotoxicity^33^. Animal studies have demonstrated PQ’s ability to induce α-synuclein upregulation, promote aggregate formation, and activate microglial^34^.

The NLR is a readily detectable inflammation marker using routine blood tests. It has been used to assess inflammatory-related lesions such as tumors, ischemic stroke, and coronary artery disease^35–37^. Neutrophils are important members of the human immune system that have gradually gained attention for their role in APP. Studies have shown that PQ can induce rapid expression of neutrophil chemoattractant proteins in bone marrow mesenchymal stem cells. Consequently, neutrophils rapidly increase in the blood and accumulate in the lungs, promoting the production of chemokines and pro-inflammatory factors (such as tumor necrosis factor α and interleukin 8) and activating the inflammatory response. Simultaneously, alveolar macrophages generated by neutrophils initiate immune responses and generate reactive oxygen species, leading to cellular nicotinamide adenine dinucleotide phosphate (reduced coenzyme II) depletion and cell membrane lipid peroxidation, thereby promoting the expression of pro-fibrotic genes in fibroblasts and resulting in pulmonary fibrosis^38,39^. Lymphocytes play a central role in regulating inflammatory responses in the human body. However, the specific mechanism through which PQ induces lymphocyte degradation remains unclear. Whether PQ inhibits the cellular immune function requires further investigation.

Our study showed that liver injury was an independent risk factor for death in patients with APP, which contradicts the findings of previous studies showing that toxic hepatitis is common after PQ exposure. Toxic hepatitis appears mild and transient in scope but is associated with higher complication rates, including respiratory and renal failure^40^.

PQ penetration into tissues and organs can cause a series of oxidative stress reactions, generating a large amount of reactive oxygen species and leading to organ damage or failure^41^. The kidney is the main excretory organ for PQ, eliminating 90% of PQ within 12–24 h. Consequently, patients with APP often present with acute kidney injury characterized by elevated creatinine^42^. In addition, renal dysfunction can significantly reduce the PQ excretion rate and increase its accumulation in the body, forming a vicious circle^43,44^. Further studies are needed to confirm whether correcting kidney injury improves prognosis.

CO_2_CP refers to the plasma CO_2_ content measured after isolating plasma from venous blood samples at room temperature and balancing it with the alveolar air of healthy people^45^. Two major acid–base disturbances, respiratory acidosis and metabolic alkalosis, both of which can result in increased CO_2_CP, are common in patients with respiratory diseases. If respiratory diseases such as chronic obstructive pulmonary diseases are excluded, the impact of the respiratory acid–base balance on CO_2_CP can be minimized^46^. In contrast, reduced CO_2_CP concentrations suggest metabolic acidosis^47^ or respiratory alkalosis^48^, both of which are indicators of poor outcomes. Most often, decreased CO_2_CP indicates the presence of metabolic acidosis; however, it could also reflect a decline in bicarbonate concentration as compensation for respiratory alkalosis. Therefore, it is considered a broad indicator of lung and kidney damage. In this study, the CO_2_CP of the mortality group was significantly lower than that of the survival group (*P* < 0.05). As the lungs and kidneys are important target organs of APP, this index can be regarded as a comprehensive evaluation index.

Considering that some primary hospitals cannot detect plasma PQ concentrations, we constructed a predictive model for plasma concentrations without PQ and compared it with SIPP. The second model showed the AUC of 0.882 (95% CI 0.853–0.911). Although the performance of the second model is not as good as that of the first, it is still worth promoting.

The current study has some limitations. First, it was a single-center retrospective study with selection bias; furthermore, only 724 patients were included because of missing data, which may limit the scalability of the model. Second, this study only included the clinical data of patients with APP at the time of enrollment and did not combine the clinical data after treatment to evaluate patient survival after discharge, which may have affected the results. Owing to limitations in clinical data, the GCS classification in this study was restricted to < 15/15. However, in future studies, we aim to collect more comprehensive data to improve nomogram precision. Moreover, because of the severe lack of clinical data and absence of indicators of lung injury, this study was performed retrospectively. The model must be updated when more multicenter data become available.

## Conclusion

We developed a predictive model for in-hospital mortality in patients with APP. The nomogram, which includes six risk factors with favorable predictive accuracy, discrimination, and clinical utility, allows for simple and rapid individual patient risk estimates. It can be used on admission to the ED to predict mortality and facilitate early risk stratification and actionable measures in clinical practice after further external validation.

## Data Availability

The datasets generated during and/or analyzed during the current study are not publicly available due to data confidentiality. However, they can be obtained from the corresponding author upon reasonable request.
